# Impact of Physical Activity and Dietary Habits on Mental Well-Being in Patients with Diabetes Mellitus

**DOI:** 10.3390/nu17061042

**Published:** 2025-03-16

**Authors:** Battamir Ulambayar, Amr Sayed Ghanem, Ágnes Tóth, Attila Csaba Nagy

**Affiliations:** 1 Department of Health Informatics, Faculty of Health Sciences, University of Debrecen, 4032 Debrecen, Hungary; ulambayar.battamir@etk.unideb.hu (B.U.); aghanem@etk.unideb.hu (A.S.G.); 2 Department of Integrative Health Sciences, Faculty of Health Sciences, University of Debrecen, 4028 Debrecen, Hungary; toth.agnes@etk.unideb.hu

**Keywords:** diabetes mellitus, mental well-being, eating habit, physical activity, WHO-5 index

## Abstract

**Background**: The prevalence of diabetes mellitus (DM) is increasing worldwide, and mental health problems such as depression, anxiety, and diabetes distress are common co-morbidities that negatively impact the quality of life, complications, and treatment outcomes of patients with DM. **Objectives**: In this study, we assessed the impact of dietary patterns and physical activity on the well-being of patients with DM. **Methods**: A cross-sectional design and data from the European Health Interview Survey (EHIS) data collected in Hungary in 2019, and determination of the relationship between dietary habits and physical activity-related variables contained in the data and the World Health Organization-Five Well-Being Index (WHO-5 index) by suitable statistical methods. **Results**: Logistic regression showed higher odds of poor mental well-being in females (OR = 1.61, 95% CI: 1.08–2.42) and lower odds with daily fruit intake (OR = 0.52, 95% CI: 0.31–0.89). Infrequent white meat (OR = 3.34, 95% CI: 1.35–8.22) and dairy intake (OR = 1.60, 95% CI: 1.18–2.64) were associated with poorer well-being. Walking 4–7 days/week reduced the odds by 57% (OR = 0.43, 95% CI: 0.23–0.83). **Conclusions**: The results demonstrate that regular exercise and the consumption of fruits, dairy products, and white meat have beneficial effects on the mental well-being of patients with DM.

## 1. Introduction

Diabetes mellitus (DM) is a major global health concern. It is most commonly classified into three types: type 1 diabetes mellitus (T1DM), type 2 diabetes mellitus (T2DM), and gestational diabetes. Among these, T2DM is the most prevalent, accounting for approximately 90–95% of individuals with DM [1,2]. Hallmarks of T2DM are insulin resistance and a relative deficiency of insulin secretion, whereas T1DM causes an absolute deficiency of beta-cell function, which is commonly due to the autoimmune destruction of beta-cells. Gestational diabetes mellitus (GDM) is defined as glucose intolerance initially identified during pregnancy. Patients with GDM are at greater risk of acquiring T2DM in the future [3]. Due to lifestyle changes associated with economic development and industrialization, the incidence of DM in the past three decades has been rising sharply. According to the World Health Organization, between 1990 and 2022, the number of people living with DM increased from 200 million to 830 million [4], causing an increase in mortality and morbidity worldwide [5].

Beyond age and genetics, modifiable risk factors including smoking, alcohol consumption, education, physical inactivity, and harmful eating patterns are the most important contributors to the development of T2DM [6,7]. Studies suggest that increased consumption of ultra-processed food, which are industrial formulations produced by extrusion, molding, and frying using ingredients such as sugars, proteins, oils, fats, starches, hydrogenated fats, modified starches, flavor enhancers, colorings and other additives—as defined by the NOVA food classification system [8]—are associated with negative health consequences. This unhealthy diet combined with sedentary behavior [9] results in weight gain and obesity [10], elevating the likelihood of T2DM [11,12]. In contrast, hypertension, obesity, physical inactivity, and heavy alcohol consumption are modifiable risk factors shared by both T2DM and dementia [13], contributing to the overall disease burden and potentially affecting mental health outcomes. In patients with DM, these risk factors, along with obesity-related complications, can exacerbate psychological distress and increase the likelihood of developing mental health disorders. Furthermore, obesity, diabetic nephropathy, diabetes-related complications, elevated C-reactive protein levels, female sex, and low social support have been associated with a higher risk of mental disorders [14], highlighting the complex interplay between physical health and mental well-being in this population. Physical activity has emerged as an effective strategy for improving mental well-being in patients with T2DM. Regular aerobic exercise can significantly enhance self-esteem and mental health in patients with DM [15]. Addressing these modifiable risk factors through lifestyle interventions may play a crucial role in improving mental health outcomes for patients with DM.

As a consequence of metabolic dysfunction, chronic hyperglycemia is a primary clinical feature of the disease, leading to an elevated risk of microvascular and macrovascular disorders [2,16] and therefore to numerous complications, including retinopathy, nephropathy, cardiovascular diseases (CVD), neuropathy, amputation, infections, and cognitive impairment, especially if undiagnosed for many years [17]. Furthermore, the diagnosis and management of DM can be a significant life stressor for individuals [18], as the burden of living with DM profoundly affects patients’ mental health and overall quality of life [5,19]. Guilt, anxiety, and concerns about self-care are some of the symptoms of diabetes distress. These symptoms can be connected to treatment regimens, food consumption, hypoglycemia, future complications, social and interpersonal connections, and interactions with medical professionals [20]. Diabetic distress is common and persistent, with an estimated prevalence of more than 20% in both T1DM and T2DM [21]. While symptoms of diabetes distress may include low mood, it is distinct from depression, which is also prevalent in individuals with DM [19]. In general, between 10% to 15% of diabetics suffer from depressive disorders, which is approximately twice as common as depression compared to people without DM [22].

The comorbidity of mental disorders substantially lowers the prognosis of the disease and increases mortality [22], underscoring the critical need for integrated treatment approaches that address both mental health and physical aspects of the condition. Managing the mental challenges associated with glucose control is essential in reducing the burden of DM and its complications [23], where maintaining a healthy weight with proper nutrition and regular exercises are key components, in which patients can actively participate [24]. While previous research has explored the individual effects of physical exercise and dietary habits on the physical health of patients with DM, there is limited literature addressing their combined impact on mental health outcomes in this population. Most studies focus on either physical activity or nutrition in isolation, overlooking the potential synergistic effects of these lifestyle factors on psychological well-being of patients. Therefore, we hypothesized in the present research that patients with DM would benefit mentally from a healthy, balanced diet and regular exercise.

This study aims to investigate the impact and highlight the importance of physical activity and dietary habits on the mental well-being of patients with DM, emphasizing that the mental well-being of individuals with DM is crucial, as the connection between physical and mental health is often underestimated. Using data from the European Health Interview Survey (EHIS) in Hungary, the findings may help in understanding the physical and mental health needs of individuals with DM, contributing to better disease management and improved quality of life. By exploring specific aspects of physical activity and diet, this research seeks to provide practical insights for improving mental health outcomes in patients with DM.

## 2. Materials and Methods

### 2.1. Study Design and Data

We conducted this study using a cross-sectional design and data collected in Hungary in 2019 as part of the European Health Interview Survey (EHIS). The data collection process was conducted under the supervision of Eurostat using a standard questionnaire, and the sample size (5603) was sufficiently representative of the entire Hungarian population [25]. Except for those without diabetes mellitus (DM) and those under the age of 18, we focused on data from 545 participants who had a confirmed diagnosis of DM.

### 2.2. Variables

The EHIS 2019 survey conducted in Hungary used the World Health Organization-Five Well-Being Index (WHO-5) questionnaire to assess the mental health well-being of the study participants. The WHO-5 index has been validated as a reliable and valid screening tool for assessing the mental health status of patients with DM [26] and for detecting depression among these patients [27]. This standard questionnaire consists of five questions, each designed to be scored from 0 to 5. According to the guidelines for processing the results of the WHO-5 questionnaire, the total score is calculated as a percentage by adding the points corresponding to the selected answers of each participant and multiplying by 4 [28]. A score of 50 or less is considered poor mental well-being, indicating that the person needs mental health care [29]. We calculated the WHO-5 index for each participant according to this guideline and divided all participants into two groups: poor and better mental well-being. Subsequently, the relationship between the WHO-5 index category and dietary variables such as fruit, vegetables, meat, milk and dairy products, sweets and desserts, and water consumption, as well as variables related to physical activity, contained in the data, was determined.

### 2.3. Statistical Analysis

The association between these variables and the WHO-5 index was assessed using a Chi-square test. A logistic regression model was created to demonstrate whether dietary habits and physical activity influence the mental health of patients with DM based on the statistically significant associations obtained by the Chi-square test. The odds ratio (OR) and 95% confidence interval (CI) are used to express the outcomes of logistic regression analysis, and a *p*-value lower than 0.05 was considered to be statistically significant. STATA IC version 18.0 was used to conduct the statistical analyses used in this analysis [30].

## 3. Results

The data used in the study included a total of 545 patients with DM, representing a prevalence of 9.7 percent. Of these, 53 percent were women and 57 percent were aged 65 or older. When assessing their mental health status using the WHO-5 questionnaire, the average score was 61 ± 23. As mentioned above, when categorizing mental health status, 30.4 percent were classified as having poor mental well-being, as shown in Table 1.

When examining the relationship between dietary habits on the mental health of patients with DM, it was observed that people who consumed more vegetables (*p* = 0.024) and fruits (0.005) in their daily diet had better mental health than those who consumed less. Similarly, an increase in the consumption of white meat (*p* = 0.004) and dairy products (*p* = 0.003) has been associated with better mental health. However, red meat consumption, daily water intake, and dessert consumption were not significantly associated with mental health status. The study participants’ mental health status and primary characteristics of physical activity were compared, and it was found that 21.2 percent of those who reported moderate physical activity had poor mental health, compared to 42.2 and 28.6 percent of those who reported light and heavy physical activity, respectively (*p* = 0.001). Increasing the number of days that people walk for more than ten minutes each week (*p* = 0.044) and doing more than ten minutes of sport exercise (*p* = 0.02) have also been shown to improve mental health (Table 2).

Logistic regression analysis results showed females had 1.61 times higher odds of having poor mental well-being compared to males (OR = 1.61, 95% CI: 1.08–2.42, *p* = 0.019). This suggests that being female is a significant risk factor for poor mental well-being, with women being approximately 61% more likely to experience poorer mental health outcomes than men. It is also worth noting that when examining the relationship between dietary patterns, physical activity, and gender among people with DM, no statistically significant differences were observed. This implies that dietary and physical activity differences may not have a significant impact on the association between gender and mental health in patients with DM, though more research is required to confirm this independence.

Participants who consumed one serving of fruit per day had 48% lower odds of poor mental well-being compared to those who did not consume fruits (OR = 0.52, 95% CI: 0.31–0.89, *p* = 0.017). However, mental well-being was not significantly different for those consuming two or more servings of fruit per day. This suggests a potential nonlinear relationship between fruit intake and mental health, where moderate consumption may be beneficial but higher levels do not provide additional advantages. Future research should explore these nonlinear patterns to better understand the complex association between dietary habits and mental well-being. Participants who consumed white meat once or less per week were 3.34 times (OR = 3.34, 95% CI: 1.35–8.22, *p* = 0.009) more likely to have poor mental health than those who consumed it four or more times per week. A similar pattern was observed for milk and dairy product consumption, with people who consumed milk and dairy products 1 serving or less per week being 1.6 (OR = 1.6, 95% CI: 1.18–2.64, *p* = 0.044) times more likely to have poor mental health than those who consumed these products 4 or more times per week. This suggests that consumption of fruits, white meat, and dairy products has a positive impact on the mental health of people with DM.

The log regression analysis found a significant association between physical activity, specifically the number of days individuals walked for at least 10 min per week, and better mental well-being. The results revealed that individuals who walked 4–7 days per week had 57% lower odds of experiencing poor mental well-being compared to those who did not engage in walking at all (OR = 0.43, 95% CI: 0.23–0.83, *p* = 0.012). This finding underscores the importance of regular physical activity, even in modest amounts, as a protective factor for mental health in patients with DM (Table 3).

## 4. Discussion

This study was conducted to determine how dietary patterns and physical activity affect the mental health of patients with DM. The results showed that certain foods, such as fruit consumption, white meat and dairy products, and regular exercise, improved the mental health of patients with DM after adjusting for age and gender.

It has been found that people with DM are at increased risk of developing mental health disorders such as depression, anxiety, and eating disorders [31]. The risk of depression in patients with T1DM is three times higher than in the general population, while the risk is twice as high in patients with T2DM [32]. Furthermore, it negatively impacts their blood sugar control [33], cardiovascular complications [34], and overall quality of life [35]. Nutrition plays a crucial role in both blood sugar control and mental well-being, with deficiencies in specific nutrients linked to the pathogenesis of mental disorders and DM [36].

Research suggests a positive association between fruit and vegetable consumption and mental well-being, including for individuals with DM [37]. Higher fruit and vegetable intake is linked to better mental health outcomes in longitudinal studies, with evidence of a dose-response relationship [38]. The nutritional benefits of essential minerals, vitamins, fiber, and antioxidants [39], as well as the gut microbiome that fiber supports [40], can account for the beneficial effects of consuming vegetables and fruits on mental health in patients with DM. In contrast, fruits provide high levels of vitamin C and flavonoids, which have been linked to improved cognitive function and reduced risk of depression [41]. The antioxidants found in fruits combat oxidative stress, which is often elevated in patients with DM and can negatively affect mental health [42]. Additionally, the act of consuming fruits can serve as a healthy coping mechanism for stress, providing a sense of control over dietary choices which can enhance self-efficacy and overall mental resilience [43]. Moreover, the social and behavioral aspects of fruit consumption cannot be overlooked. Engaging in healthy eating behaviors, such as incorporating fruits into one’s diet, often occurs alongside other positive lifestyle changes, including increased physical activity and better stress management practices [44]. These lifestyle modifications contribute to improved mental health outcomes. While our results did not show a direct link between vegetable consumption and mental health in DM patients, fruit consumption had a significant positive impact, aligning with existing literature.

White meat consumption is considered a relatively healthy intake for patients with DM compared to red meat and processed meat and is therefore thought to have a beneficial effect on reducing the risk of CVD in patients with DM [45]. This protective effect is due to the low saturated fat and sodium content of white meat, which contributes to improved glycemic control and overall metabolic health [46]. Moreover, the nutritional profile of white meat includes essential nutrients that are vital for mental health. For instance, white meat is an alternative source of certain molecules, which is important for overall brain function and development [47]. Furthermore, white meat consumption reduces psychological distress in overweight or obese people, suggesting that this type of dietary choice may play an important role in managing mental health [48]. On the other hand, it is worth noting that this protective effect of white meat may not be the result of white meat consumption alone, but is also the effect of a balanced diet combined with other healthy foods [49]. In particular, the Mediterranean diet, characterized by high intake of fruits, vegetables, whole grains, legumes, nuts, fish, white meats, and olive oil, is considered one of the healthiest dietary models globally, and it can significantly improve glycemic control in patients with T2DM by reducing blood glucose levels, HbA1c, and insulin resistance [50]. Therefore, these types of healthy diets may have a positive impact on mental health in patients with T2DM by improving blood sugar control and reducing complications. Our study results show that low consumption of white meat is associated with an increased risk of poor mental health, which is consistent with the previous literature.

Dairy products and the mental health of patients with DM have a complex relationship that includes both psychological and physiological aspects. Research indicates that dairy consumption can have beneficial effects on metabolic health, which is particularly relevant for individuals with DM. For instance, studies have shown that the intake of low-fat fermented dairy products is inversely associated with the risk of developing T2DM [51]. Milk proteins can lower postprandial glucose levels by modifying physiological processes, including delayed gastric emptying and enhanced incretin and insulin responses [52]. Certain dairy components, such as leucine and calcium-regulated peptides, may alter mitochondrial function, promote beneficial gut microbial shifts, and influence inflammation and cardiovascular function, potentially linking dairy intake with lower risk of T2DM [53]. This is important because patients with DM frequently experience psychological distress associated with their condition, as mentioned above, and better metabolic control can result in better mental health outcomes [54]. Also, dairy products enriched with probiotics, prebiotics, and synbiotics have shown potential in reducing hyperglycemia, depressive behaviors, and oxidative stress in patients with DM [55]. Moreover, the consumption of dairy products, particularly those that are fermented, has been linked to various health benefits, including enhanced gut health and immune system modulation, which can indirectly influence mental well-being [56]. The psychological benefits of dairy consumption may also stem from its nutritional profile, which includes essential nutrients that support brain health [57]. However, depending on the composition of dairy products, such as those high in fat and protein, consumption of dairy products may worsen depressive symptoms [58]. Therefore, it is crucial to note that its subtypes should be considered when evaluating the effect of milk and dairy products on the mental health of DM. Our study results showed that dairy consumption positively impacts the mental health of patients with DM, suggesting that effective management of DM, including dietary changes such as the inclusion of dairy products, can lead to improved mental health outcomes.

In addition to dietary habits, physical activity is vital for blood sugar control [59] and psychological well-being [60] in patients with DM. A recent randomized controlled clinical trial found that exercise not only effectively reduces depression in patients with diabetes but also improves blood sugar control [15]. Exercise training enhances glycolipid metabolism and insulin sensitivity and modulates DNA methylation in patients with T2DM [61]. Various forms of exercise, including aerobic and resistance training, enhance cognitive function and mental health in T2DM patients, and it can be explained by the action of irisin, a myokine produced during exercise. It has been identified as a potential mechanism for improving cardiovascular and mental health in patients with T2DM [62]. Light and moderate exercise are associated with better cognitive function scores, potentially offsetting the negative impact of diabetes on cognition. This may be because exercise not only reduces the symptoms of DM by improving blood sugar control in patients with DM but also reduces symptoms of mental disorders such as anxiety, insomnia, and depression by preventing complications and improving brain function [63]. Furthermore, the benefits may be attributed to physiological changes, such as improved functioning of the hypothalamus–pituitary–adrenal axis [64]. Our study results also highlight the importance of exercise in maintaining blood sugar control and mental health in patients with DM.

Self-reported questionnaires were used to collect data for this study, which may have introduced bias due to recall inaccuracies, social desirability, or misreporting. Participants may overestimate or underestimate their dietary intake and physical activity levels, leading to potential measurement errors. Another limitation of this study is its cross-sectional design, which prevents the establishment of causal relationships between dietary patterns, physical activity, and mental well-being. Additionally, the study missed questions about the consumption of subtypes of certain food products, like dairy products, which made it challenging to estimate their effects accurately. The data used in the study did not include variables such as diabetes type, treatment, and complications, limiting the ability to account for their influence on the observed relationships. However, the main strength of this study is that we used advanced statistical methods such as logistic regression to analyze robust data collected through a Eurostat-validated questionnaire, which is representative of the Hungarian population, in addition to the use of the WHO-5 questionnaire, which has demonstrated excellent internal consistency, test-retest reliability, and unidimensional factorial validity. It is also important to note that we included most of the food categories in the balanced food pyramid to assess the impact of dietary habits on the mental health of patients with DM.

## 5. Conclusions

The results of this study demonstrate the important role that physical activity and dietary habits play in supporting the mental health of individuals with DM. In particular, it was found that regular exercise and consumption of fruits, dairy products, and white meat have beneficial effects on the mental well-being of patients with DM independent of gender and age group. However, it is worth noting that the results of this study were conducted using a cross-sectional design, and longitudinal studies are needed to confirm the observed relationships.

Based on the results of this study, we recommend the public health programs that prioritize routine mental health screenings for women with DM and implement tailored psychosocial support to mitigate this increased risk. The observed association between fruit, white meat, and dairy product consumption and better mental well-being suggests that encouraging balanced dietary patterns could be beneficial for mental health in patients with DM. Diabetes management programs should actively promote low-intensity, accessible forms of exercise, such as regular walking, which may be easier for patients to adopt and sustain. This aligns with existing physical activity guidelines but reinforces the importance of frequent, modest activity for mental health benefits. Our findings support the integration of mental health considerations into diabetes care protocols. Public health strategies should encourage multidisciplinary approaches, combining medical, nutritional, and psychological support to enhance overall well-being.

## Figures and Tables

**Table 1:** Demographic data and WHO-5 Wellbeing Index.

Variables	Category	N (%)
Age group	18–35 years old	22 (4.0)
	35–65 years old	212 (38.9)
	Older than 65	311 (57.1)
Gender	Female	288 (53.1)
	Male	254 (46.9)
WHO-5 Wellbeing Index	Poor mental well-being (≤50)	165 (30.4)
	Better mental well-being (>50)	377 (69.6)

**Table 2:** Associations of WHO-5 Wellbeing Index with eating habits and physical activity.

Variables	Category	WHO-5 Wellbeing Index		*p* Value
		Poor (≤50)	Better (>50)	
Vegetable consumption per day	No vegetable consumption	57 (37.5)	95 (62.5)	**0.024**
	1 serving	52 (34.0)	101 (66.0)	
	2 servings	39 (24.7)	119 (75.3)	
	More than 3 servings	16 (21.9)	57 (78.1)	
Fruit consumption per day	No fruits consumption	97 (37.6)	161 (62.4)	**0.005**
	1 serving	33 (23.2)	109 (76.8)	
	2 servings	22 (23.4)	72 (76.6)	
	More than 3 servings	9 (23.1)	30 (76.9)	
Drinking water per day	2 L and more	77 (26.3)	216 (73.7)	0.068
	1–2 L	52 (35.9)	93 (64.1)	
	Less than 1 L	35 (35.0)	65 (65.5)	
Sweets and biscuit consumption	Never eats	41 (75.9)	13 (24.1)	0.293
	Not regular, only occasions	205 (66.4)	104 (33.6)	
	Less than one a day	65 (71.4)	26 (28.6)	
	More than one a day	62 (74.7)	21 (25.3)	
Red meat consumption	More than 4 times a week	22 (33.3)	44 (66.7)	0.086
	1–3 times a week	86 (27.1)	232 (72.9)	
	Less than 1 time a week	56 (36.8)	96 (63.1)	
White meat consumption	More than 4 times a week	25 (21.7)	90 (78.3)	**0.004**
	1–3 times a week	122 (31.4)	267 (68.6)	
	Less than 1 time a week	16 (51.6)	15 (48.4)	
Milk and milk product consumption	More than 4 times a week	95 (28.4)	240 (71.6)	**0.003**
	1–3 times a week	26 (24.5)	80 (75.5)	
	Less than 1 time a week	53 (55.2)	43 (44.8)	
Primary characteristics of physical activity	Light	98 (42.2)	134 (57.8)	**0.001**
	Moderate	61 (21.2)	226 (78.8)	
	Heavy	4 (28.6)	10 (71.4)	
Number of days walked at least 10 min a week	Did not walk	133 (33.0)	270 (67.0)	**0.044**
	1–3 days	17 (28.3)	43 (71.7)	
	4–7 days	15 (18.9)	64 (80.1)	
Number of days engaged in sport for at least 10 min per week	Did not engage in sport	139 (33.4)	277 (66.6)	**0.02**
	1–3 days	17 (22.7)	58 (77.3)	
	4–7 days	9 (17.6)	42 (82.4)	

Bold values indicate statistical significance (*p* < 0.05) based on Pearson’s chi-squared test.

**Table 3:** Logistic regression model.

Variables		OR (95%CI)	*p*-Value
Gender	Male (Reference)		
	Female	**1.61 (1.08–2.42)**	**0.019**
Age group	18–35 years old (Reference)		
	35–65 years old	1.89 (0.56–6.31)	0.299
	Older than 65	1.53 (0.45–5.12)	0.487
Vegetable consumption per day	No vegetable consumption (Reference)		
	1 serving	1.08 (0.62–1.86)	0.774
	2 servings	0.7 (0.39–1.23)	0.222
	More than 3 servings	0.63 (0.3–1.31)	0.222
Fruit consumption per day	No fruit consumption (Reference)		
	1 serving	**0.52 (0.31–0.89)**	**0.017**
	2 servings	0.62 (0.34–1.13)	0.122
	More than 3 servings	0.62 (0.25–1.48)	0.258
White meat consumption	More than 4 times a week (Reference)		
	More than 1–3 times a week	1.48 (0.88–2.5)	0.133
	Less than 1 time a week	**3.34 (1.35–8.22)**	**0.009**
Milk and milk product consumption	More than 4 times a week (Reference)		
	More than 1–3 times a week	0.67 (0.39–1.14)	0.145
	Less than 1 time a week	**1.6 (1.18–2.64)**	**0.044**
Number of days walked at least 10 min a week	Did not walk 10 min (Reference)		
	1–3 days	0.97 (0.51–1.85)	0.939
	4–7 days	**0.43 (0.23–0.83)**	**0.012**

Bold values indicate statistical significance (*p* < 0.05) Odds ratios are adjusted for variables in the model.

## Data Availability

The data analyzed in this study are subject to the following licenses/restrictions: The data presented in this study are available upon request from the Hungarian Central Statistical Office, which performed and supervised the data collection. Requests to access these datasets should be directed to the Hungarian Central Statistical Office, www.ksh.hu/?lang=en (accessed on 15 February 2025).
